# Electrical discharges in water induce spores’ DNA damage

**DOI:** 10.1371/journal.pone.0201448

**Published:** 2018-08-13

**Authors:** Camille Lamarche, Charlotte Da Silva, Gauthier Demol, Etienne Dague, Marie-Pierre Rols, Flavien Pillet

**Affiliations:** 1 ITHPP-Alcen, Hameau de Drèle, Thégra, France; 2 Institut de Pharmacologie et Biologie Structurale, IPBS, Université de Toulouse, CNRS, UPS, Toulouse, France; 3 LAAS-CNRS, Université de Toulouse, CNRS, Toulouse, France; University of Connecticut, UNITED STATES

## Abstract

Bacterial spores are one of the most resilient life forms on earth and are involved in many human diseases, such as infectious diarrhea, fatal paralytic illnesses and respiratory infections. Here, we investigated the mechanisms involved in the death of *Bacillus pumilus* spores after exposure to electric arcs in water. Cutting-edge microscopies at the nanoscale did not reveal any structural disorganization of spores exposed to electric arcs. This result suggested the absence of physical destruction by a propagating shock wave or an exposure to an electric field. However, Pulsed-Field Gel Electrophoresis (PFGE) revealed genomic DNA damage induced by UV radiation and Reactive Oxygen Species (ROS). UV induced single-strand DNA breaks and thymine dimers while ROS were mainly involved in base excision. Our findings revealed a correlation between DNA damage and the treatment of spores with electrical discharges.

## Introduction

Since their discovery by Koch in 1884 [1], pathogenic spores have been identified as responsible for many diseases, such as lethal respiratory infections [2,3], fatal paralytic illnesses (botulism) [4,5] and infectious diarrhea. Within this context, spore inactivation raises a grave issue to human health. The major obstacle is the extraordinary resistance of spores to chemical, environmental and physical stresses. Dormant spores are able to survive in space [6] and during geological time spans [7]. Such resistance can be explained by the presence, inside the core, of α/β-type small, acid-soluble spore proteins (SASPs). These proteins saturate DNA which adopts an A-like conformation. The consequence is the creation of a thymidyl-thymidine adduct termed spore photoproduct (SP) under UV radiation (254 nm) instead of cyclobutane pyrimidine dimers (CPDs) and (6–4)-photoproducts (64PPs). Furthermore, the presence of a high level of Ca^2+^-dipicolinic acid (DPA) is responsible for the lack of metabolism and the low amount of water in the spore’s core. Consequently, a low amount of reactive oxygen species can be generated [8,9]. These two components thus play a major role in DNA protection against UV radiation and oxidizing radicals. The extreme resistance of spores is also supported by the spores’ outer layers. In effect, the latter consists of a highly impermeable inner membrane, a peptidoglycan cortex, an outer membrane and a protein coat [10].

Within this context, spore inactivation is a huge challenge and different strategies have been developed for spore inactivation. Decontamination of water commonly uses chemical compounds, such as calcium hypochlorite and chlorine dioxide [11]. However, these chemical compounds are unsafe and require special handling. Therefore, other strategies should be investigated.

Electric arc technology seems to be a promising alternative for spore killing. During the exposure to electric arcs, many phenomena may be involved in bacterial death: ultraviolet (UV) radiation [12], oxidative stress [13], shock waves [14] and electric field [15]. The main phenomena which inactivate bacteria are UV radiation and oxidizing radicals [16,17]. UV radiation are known to induce strand breaks and pyrimidine dimers in *Bacillus subtilis* spore DNA [18]. Hydrogen peroxide induces a lack of metabolism during spore germination [19,20]. While, shock waves and electric field disorganize the spore structure [21]. However, the relative importance of each phenomenon during spore inactivation by electric arcs remains undescribed.

For this, we focused on the DNA integrity and the spore structure after the exposure to electric arcs in water. Genomic DNA integrity was evaluated by Pulsed-Field Gel Electrophoresis (PFGE) and endonucleases digestions. Spore structure integrity was addressed at the nanoscale by cutting-edge electron microscopies and multiparametric Atomic Force Microscopy (AFM) in living conditions [22,23]. Our results revealed the absence of physical damage including morphology and roughness. By contrast, we observed the presence of DNA damage due to UV radiation and the production of Reactive Oxygen Species (ROS).

## Material and methods

### Growth conditions and sporulation

The *Bacillus pumilus* strain (ATCC 27142), a non-pathogenic model for radiation treatment [24,25], was used for this work.

For the vegetative experiment, two Petri dishes were spread with 100 μL of *Bacillus pumilus* suspension and placed for 16 h at 37°C. To pick up bacteria, 1 mL of distilled water was put on Petri dishes. This action was repeated four times per dish.

To obtain spores, vegetative bacteria were cultivated overnight at 37°C in Luria broth (Sigma-Aldrich, France). Afterwards, to induce sporulation, the cultivated vegetative bacteria were diluted by a factor of 1:1000 times in Difco Sporulation Medium [26] and incubated at 37°C for 5 days under an agitation rate of 200 rpm. To kill the residual vegetative bacteria, the culture was incubated at 80°C for 20 min. Spore suspension was collected after multiple cycles of centrifugation (10,000 × g, 5 min) and kept in deionized water at 4°C prior to the experiment. Spore preparations consisted of single spores (>97%), as observed by phase-contrast microscopy.

### Electric arcs exposure

Vegetative bacteria were diluted at 0D_600nm_ = 0.02 and spores at 0D_600nm_ = 0.05 in the 8 liters of the solution exposed.

The electric arcs in water were obtained by high voltage discharges delivered by a Marx generator. The principle of electric arcs generation is described in S1 Fig. The parameters used in this experiment were a discharged voltage of 95 kV with a pulse repetition frequency of 10 Hz. Every 0.1 s, a damped signal with a period of 3.3 μs and a total duration of 30 μs was produced.

### Evaluation of the inactivation rate

Following electric arcs exposure, spores were diluted, spread on Plate Count Agar (PCA) medium and incubated overnight at 37°C. The Eq (1) was used to determine the inactivation rate.

$$IR=\text{log}\;10\left(\frac{NO}{N}\right)$$ (1)

IR inactivation rate

N0 initial microbial concentration

N number of survivors after the treatment

For each condition tested, 3 independent experiments were carried out with a total of 9 Petri dishes per analysis.

### Evaluation of Reactive Oxygen Species (ROS) effect

To neutralize the effects of the ROS formation during electric arcs exposure, 1 mM of pyruvate (Sigma-Aldrich, France) was added in the solution. Pyruvate is known to induce a passive protection against ROS [27]. Three independent experiments were carried out.

### Evaluation of UVs effect

To quantify the number of photons emitted from the electrical discharges, we used a chemical actinometer (potassium ferrioxalate), a photon-sensitive compound.

A spectrophotometer cuvette (38.5 mm x 1 mm x 1 mm) was placed above the electrode at 115.89 mm. The optical depth of this actinometer is approximately 2 cm. Consequently, as the photon going through the cuvette had a path of 1 cm, aluminum paper was placed on the other side to reflect UV rays. For each experiment, 40 μL of 6 mM potassium ferrioxalate was pipetted on the Quartz spectrophotometer cuvette and added to 40 μL of 0.1% (w/v) 1,10-phenanthroline, 20 μL of sodium acetate buffer (0.5 M acetate sodium with 0.0375% (w/v) 1,10-phenanthroline and 900 μL of distilled water. After 30 minutes at room temperature, the 0D_510nm_ was measured. The blank was made with a sample not submitted to the electrical treatment. The number of pulses used was: 100, 250, 400 and 500 pulses at 10 Hz. The quantity of Fe^2+^ produced was calculated from the graph obtained with Fe(SO_4_)_2_(NH_4_)_2_. It was determined that 4.1 x 10^−7^ moles of ferrous iron in 3.6 mL test solution produced an absorbance of 1.0 at 510 nm in a 1 cm optical path. Because of the surface of Quartz cuvette crossed by the photon (385 mm^2^) and the distance from the electrical discharge (115.89 mm), the fraction of photons absorbed by the cuvette was 1:110 in comparison to the total amount of light produced. To calculate the photons from the Fe^2+^ production, a quantum yield of 1.26 was used [12,28]. The relation found to define the number of photons produced in function of the absorbance measured at 510 nm was the following for A = 1.0:

$$\left(4.1*10^{-7}\right)N_{A}\frac{110}{1.26}=2.1*10^{19\;}photons$$ (2)

*N*_*A*_ the Avogadro’s number.

The number of photons created in function of the number of electric arcs was shown in (S2 Fig). In a second experiment, 3.6 mL of spore suspension with an OD_600 nm_ = 0.05 were placed in a Quartz vessel in order to fill the cuvette. After each set of pulses, the distilled water present in the vessel was changed in order to avoid the accumulation of metallic particles that come from electrodes erosion. Three independent experiments were carried out.

### Electron microscopy experiments

In order to observe cell damage by electron microscopy, spores were fixed with 2% of glutaraldehyde in 0.1 M Sorensen phosphate buffer at pH 7.2. The samples were processed by the CMEAB platform (Toulouse, France). For Scanning Electron Microscopy (SEM), images were visualized with an electron microscope Quanta^™^ 250 FEG (FEI, USA) at an accelerating voltage of 15 kV. For Transmission Electron Microscopy (TEM), images were collected with an HT 7700 (Hitachi, USA) at 80 kV.

### AFM experiments

Spores were immobilized for 1 h on a glass slide functionalized with polyethylenimine (Sigma-Aldrich, France) as described previously [29]. Spores were kept under 4.1 mM NaCl during the experiments. AFM measurements were performed in liquid using the Quantitative Imaging mode [22] with a Nanowizard III (JPK Instruments, Germany) and MLCT cantilevers (Bruker, Germany) with a measured spring constant at around 0.5 N/m and a force applied of 10 nN.

### Statistical analysis

The Student’s t-test was used to perform statistical analysis.

### DNA analysis

#### Spore decoating

Spore coat removal was performed from 1 mL of irradiated or control suspensions at OD_600nm_ of ~ 10, as previously described in the literature [30,31]. Briefly, after centrifugation for 5 min at 12,000 × g, the pellets were resuspended twice in 500 μL of fresh decoating solution (50 mM Tris-HCl [pH 8]; 8 M urea; 50 mM dithiothreitol; 1% sodium dodecylsulfate; 10 mM EDTA) and incubated for 45 min at 37°C. The decoated spores were washed 5 times in 1 mL TES buffer (10 mM Tris-HCl [pH 8]; 10 mM EDTA; 150 mM NaCl).

#### DNA extraction

Chromosomal DNA was extracted with a modified protocol from Otlewska *et al*. [32]. The decoated spore suspensions were adjusted to OD_600nm_ of 3 in 1 mL of TES buffer, centrifuged and resuspended in 200 μL of lysis buffer (50 mM Tris-HCl [pH 8]; 50 mM EDTA [pH 8]; 0.5% Tween 20; 0.5% Triton X-100) containing 4 mg/mL lysozyme (Sigma-Aldrich, France) and 0.4 mg/mL RNase A (Euromedex, France). An equal volume of 2% CleanCut Agarose (Bio-Rad, France) was immediately added to the mixture and 90 μL were dispensed into a plug mold (Bio-Rad). After solidification, the plugs were incubated in 1 mL lysis buffer containing 2 mg/mL lysozyme and 0.2 mg/mL RNase A at 37°C for 2 h. The plugs were then rinsed with 1 mL TE buffer (10 mM Tris-HCl; 1 mM EDTA; pH 8) and incubated overnight at 50°C in 1 mL lysis buffer supplemented with 1 mg/mL proteinase K (Promega, France) and 350 μL deproteinization buffer (3 M guanidine HCl; 20% Tween 20). The plugs were washed once in TE buffer at room temperature, once in TE buffer containing 1 mM Pefabloc SC (Roche, France) at 37°C and twice in TE buffer at room temperature, for 1 h each.

#### Detection of base excisions and pyrimidine dimers

For each condition, a plug slice of 4 × 2 mm was equilibrated with 150 μL of the 1x endonuclease buffer, provided by the manufacturer, at 37°C for 30 min. To detect apurinic/apyrimidinic sites and pyrimidine dimers, DNA was digested respectively with 5 U of endonuclease IV or T4 endonuclease V (New England Biolabs, France), in 150 μL of fresh buffer for 1 h at 37°C.

#### PFGE analysis

The previous endonuclease-digested plugs were equilibrated with 150 μL of the 1× *NotI* buffer, provided by the manufacturer, for 30 min at 37°C. DNA was then digested with 15 U of *Not*I (Thermo Scientific, France) in 150 μL of fresh restriction buffer overnight at 37°C. To detect single-strand breaks generated directly by electric arcs or following the endonuclease IV or T4 endonuclease V digestion, DNA was pre-incubated in 300 μL of the 1× S1 nuclease buffer for 30 min at 37°C and digested with 3 U of S1 nuclease (Thermo Scientific) in 300 μL of fresh buffer for 1 h at 37°C (S3 Fig). Plugs were washed 4 times in TE buffer for 30 min at room temperature. DNA fragments were separated on a 1% pulsed field certified agarose gel (Bio-Rad) in 0.5× TBE buffer (45 mM Tris base; 45 mM boric acid; 1 mM EDTA). Electrophoresis was carried out in a CHEF-DR III system (Bio-Rad) at 14°C for 18 h at 6V/cm with the pulse time increasing from 1 to 50 s and at an included angle of 120°. Lambda concatemers (48.5–1,000 kb; Bio Rad) were used as molecular weight standards. The gel was stained in 0.5 μg/mL ethidium bromide solution for 30 min, rinsed for 40 min in distilled water and revealed under UV illumination using the Gel Doc XR transilluminator (Bio-Rad).

## Results

### Electric arcs induce spore inactivation

**Fig 1:**
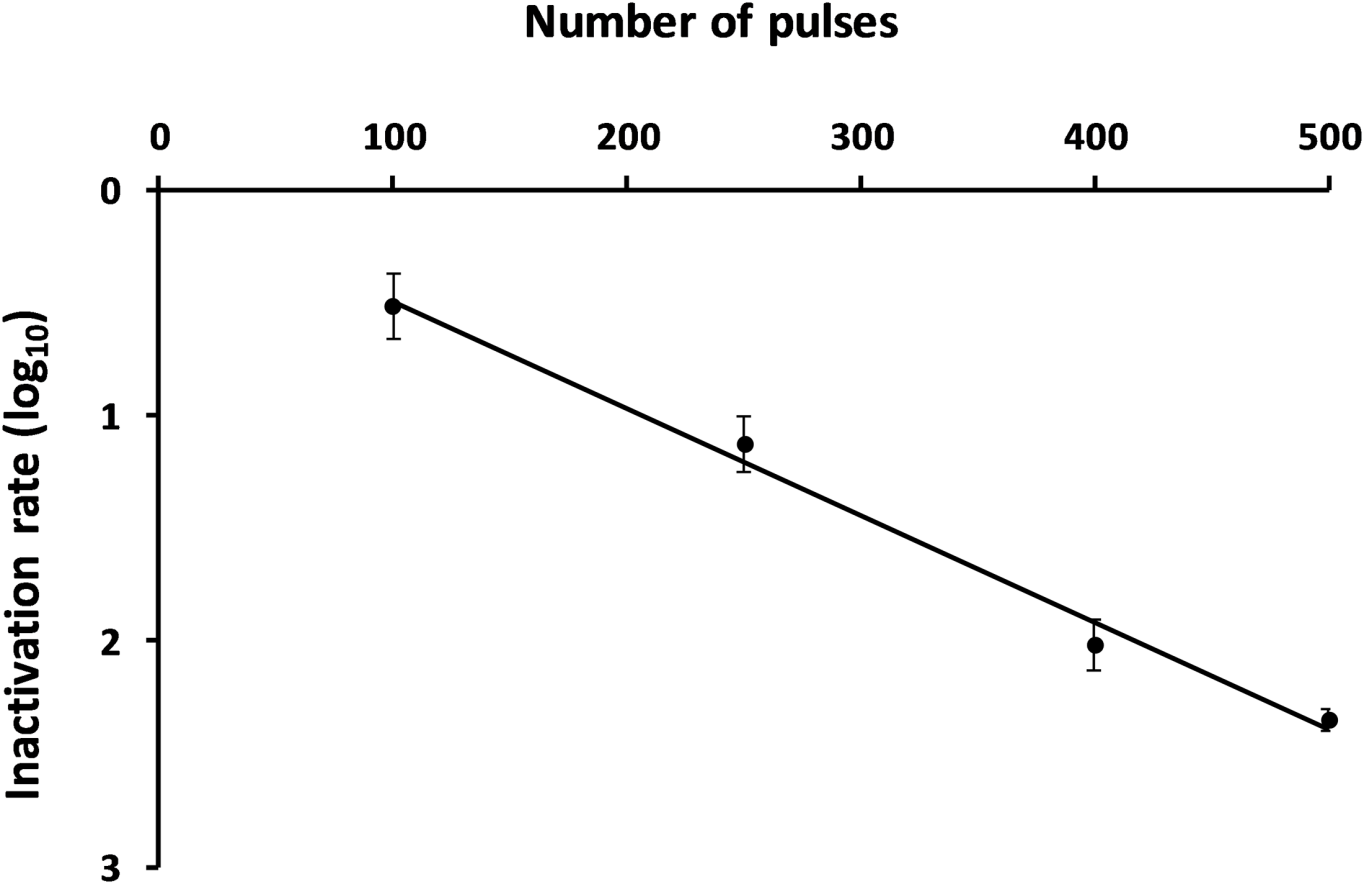
Influence of the number of electric arcs on spore inactivation. After 500 electric arc pulses, an inactivation rate of 2.2 log_10_ ± 0.2 (99%) was found from 3 independent experiments.

Spores of *Bacillus pumilus*, at OD_600nm_ of 0.05 (10^7^ CFU/mL), were exposed to different number of pulses: 100, 250, 400 and 500 electric arcs at a repetition frequency of 10 Hz (Fig 1). The inactivation rate was determined from 3 independent experiments by colony counting calculated from untreated spores. In accordance with our objectives, an inactivation rate of 2.2 log_10_ ± 0.2 (99%) was found in distilled water after 500 pulses. This result confirmed the efficiency of electric arcs to induce bacterial spore inactivation in water.

Spores of *Bacillus pumilus* were diluted in distilled water with 1 mM pyruvate at OD_600nm_ of 0.05 (10^7^ CFU/mL) and exposed to 500 electric arcs at a repetition frequency of 10 Hz. It was shown that the efficiency of the treatment was decreased. In this condition, the inactivation rate was 1.78 log_10_ ± 0.2 (98%) (S4 Fig). ROS play a role in spore inactivation.

Furthermore, spores of *Bacillus pumilus* were placed in a Quartz cuvette in order to isolate UV phenomena (without ROS, shock waves and electric field). After 500 electrics arcs, an inactivation rate of 0.72 log_10_ ± 0.08 (81%) was found (S4 Fig). UVs alone were also involved in spore inactivation.

### Spore inactivation is not due to spore structure damage

The spore wall is crucial for the spore survival. Therefore, we decided to explore the integrity of the spore structure after exposure to electric arcs. We used different microscopy techniques such as Scanning Electron Microscopy (SEM), Transmission Electron Microscopy (TEM) and Atomic Force Microscopy (AFM) to get a global, local and biophysical picture of the bacterial interface. SEM gave information at the cell scale, while TEM made it possible to explore, in depth, the spore structure. Finally, AFM experiments, conducted in liquid, provided valuable data on the spores’ outer layers (roughness, topography) and biophysical properties (rigidity, elasticity).

**Fig 2:**
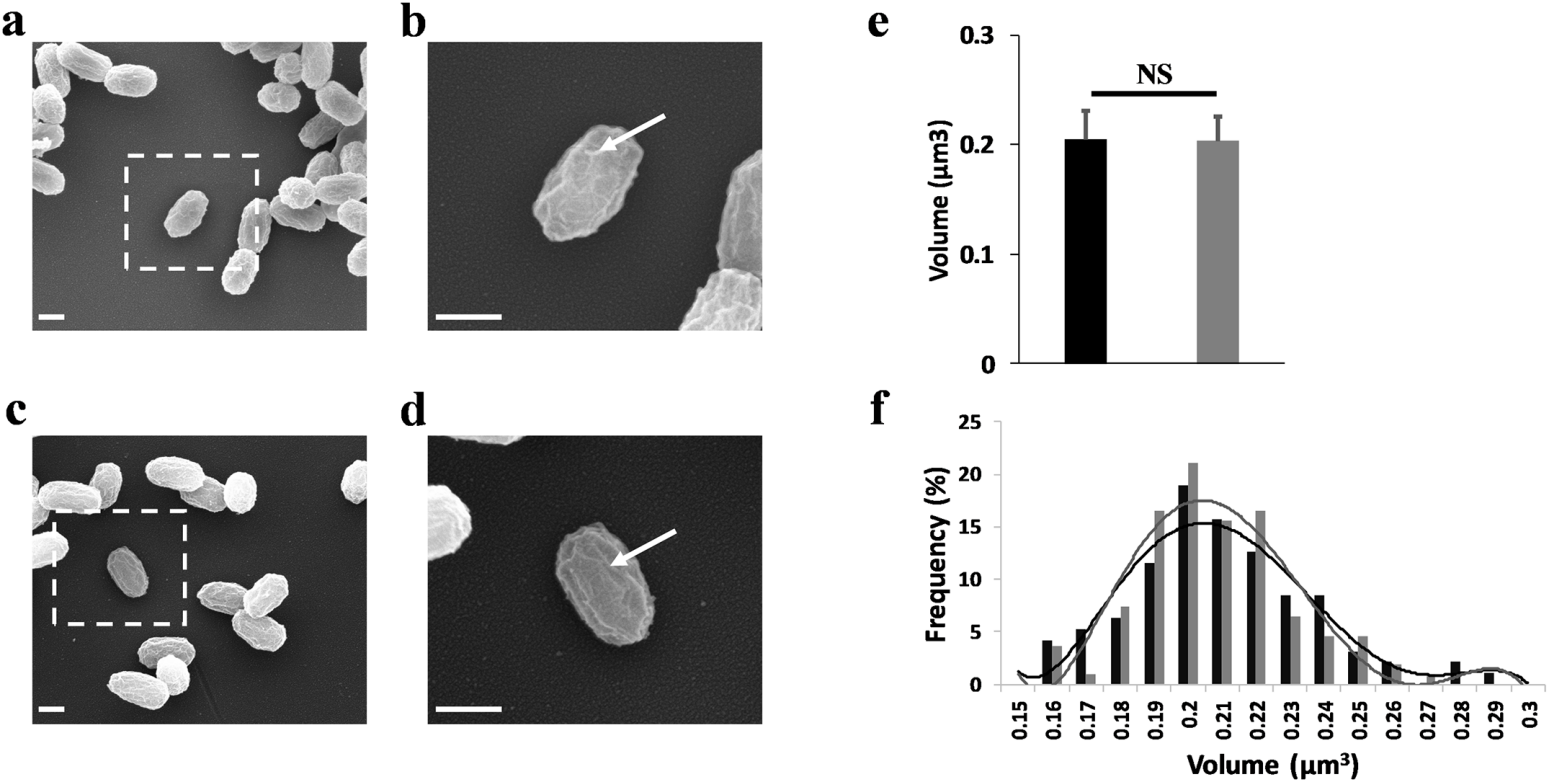
Electric arcs do not induce morphological damage on spores. **(a)** SEM image showing a population of untreated spores. Inset shows the location of a zoomed individual spore. **(b)** High resolution image of a control spore. The white arrow exhibited the protein ridges on spore coat surface. **(c)** Exposed spores visualized by SEM image after 500 electric arcs exposure. **(d)** High resolution image of an exposed spore. The electric arcs did not induce disruption of protein ridges (white arrow). Scale bars: 500 nm. **(e)** Statistical analysis of the spore volume. No variation was observed between untreated and exposed spores. **(f)** Frequency distribution of the volume of spores in control condition (black bars) and spores exposed (grey bars). Lines indicated the fitted gauss curves. The volumes were calculated from 100 spores in both conditions.

We first explored by SEM the global morphology of spores and the coat surface (Fig 2). Images showed homogenous populations in control condition (Fig 2a) and after exposure (Fig 2c). The calculated average volume was not statistically different with 0.20 ± 0.03 μm^3^ for unexposed spores and 0.20 ± 0.02 μm^3^ for treated spores (Fig 2e). The diagram in Fig 2f confirmed these observations, with a frequency distribution of spore volume similar in both conditions. Furthermore, high-resolution images in both conditions (Fig 2b and 2d) revealed the presence of nanostructures on the spore surfaces. These structures are classically described in the literature, as protein ridges present on the spore coat surface in *Bacillus* species [33]. All these results suggested a non-alteration of the spore morphology after eradication by electric arcs.

**Fig 3:**
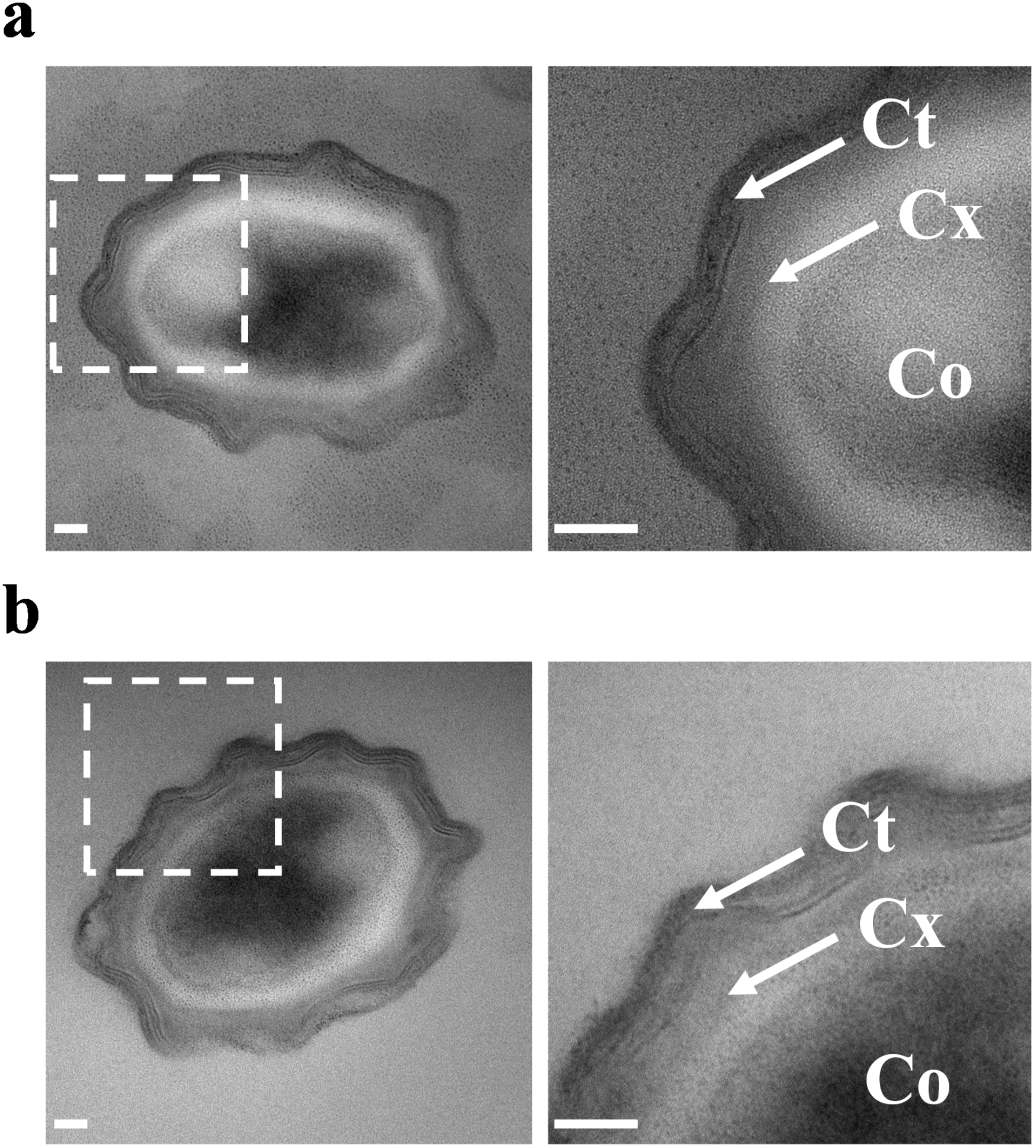
Electric arcs do not lead to the destruction of the spore structure. **(a)** Example of TEM image of an untreated spore. Inset revealed the structure of the spores at the nanoscale. The core (Co), the cortex (Cx) and the coat (Ct) are visualized. **(b)** TEM image of a typical spore exposed to electric arcs. The spore structure is similar to that of untreated spore. Scale bars: 50 nm. At least, 10 spores were imaged for each condition and one representative image was shown.

We then explored by TEM the entire spore structure (Fig 3). As expected for *Bacillus pumilus* spores [21], the coat, the cortex and the core were perfectly distinguished in untreated spores (Fig 3a). After electric arcs, the ultrastructure of spores was maintained (Fig 3b). These observations supported the hypothesis of a non-physical disruption of spore structure during inactivation by electric arcs. However, the structural organization of decoated spores were affected by electric arcs exposure (Data not shown). This result confirmed the importance of the coat in spore resistance. On the contrary, as TEM images revealed a cytoplasm content leakage, a loss of the cell wall integrity was suggested in vegetative bacteria after electric arcs treatment (S5 Fig).

**Fig 4:**
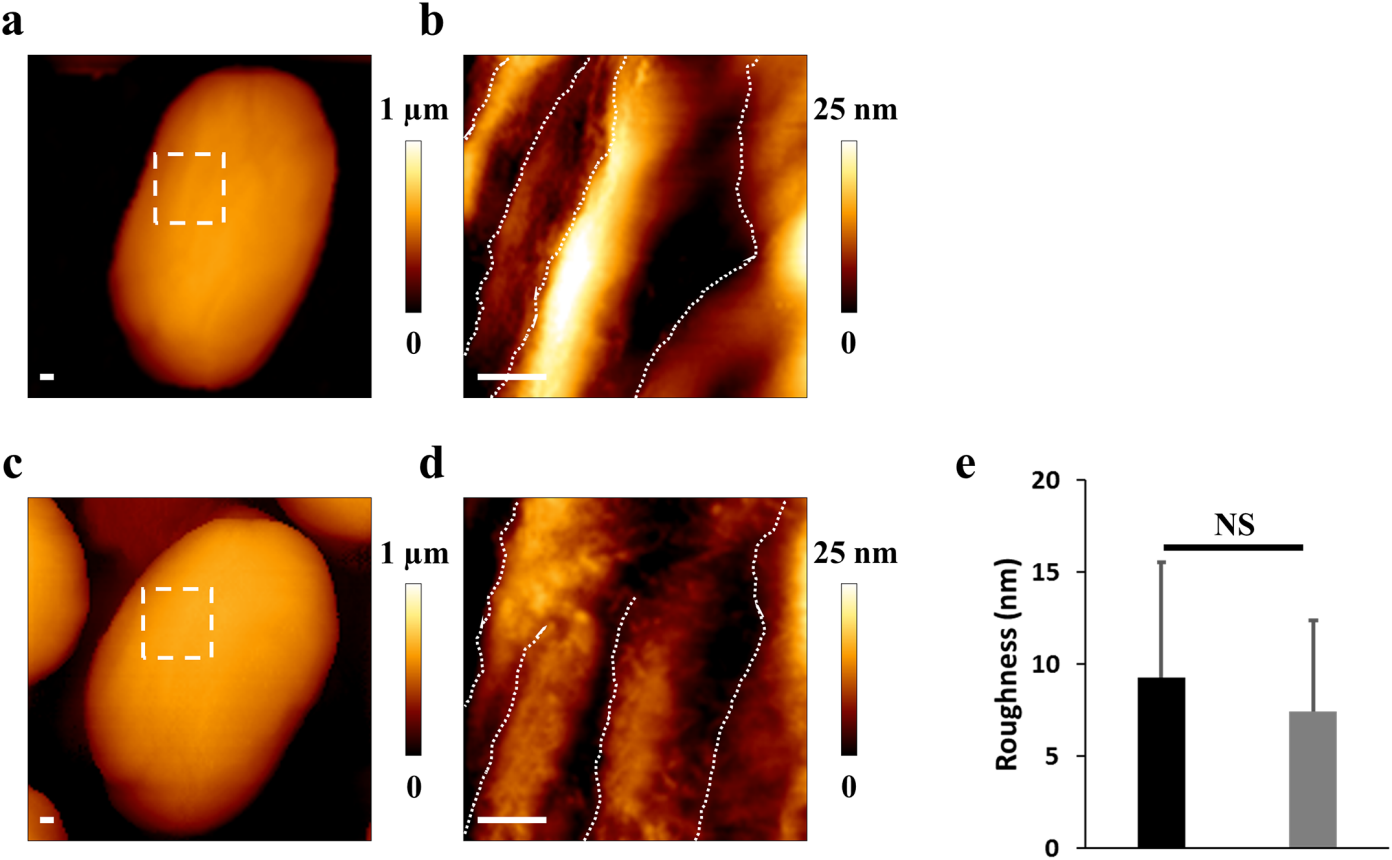
AFM in liquid does not reveal modification of volume and roughness. **(a)** Height AFM image of an untreated spore. Inset reveals the location of the zoomed section. **(b)** High resolution image exhibiting the protein ridges on the surface (white dotted lines). **(c)** Example of an exposed spore. **(d)** The spore surface was similar to the control spore. Scale bars: 100 nm. **(e)** Statistical analysis of the roughness performed from 15 spores in each condition. The roughness was comparable in both conditions.

AFM in liquid were performed to study the spore surface without chemical treatment or fixation, to preserve the integrity of the studied microorganism [23]. Examples of typical spores were imaged by AFM and shown in Fig 4. On the untreated spore (Fig 4a), the high resolution image revealed protein ridges, as previously described [21]. After electric arcs treatment (Fig 4b), the protein ridges were visible and confirmed the previous observations obtained by SEM. To quantify the surface roughness, 15 height AFM images of individual spores were analyzed for each condition. The roughness analyzed was 9 ± 6 nm for control spores and 7 ± 6 nm on treated spores. The statistical analysis revealed no significant differences. These results were correlated with electron microscopy and confirmed the non-disruption of the spores’ outer layers during inactivation by electric arcs.

### DNA damage were observed after electric arcs exposure

**Fig 5:**
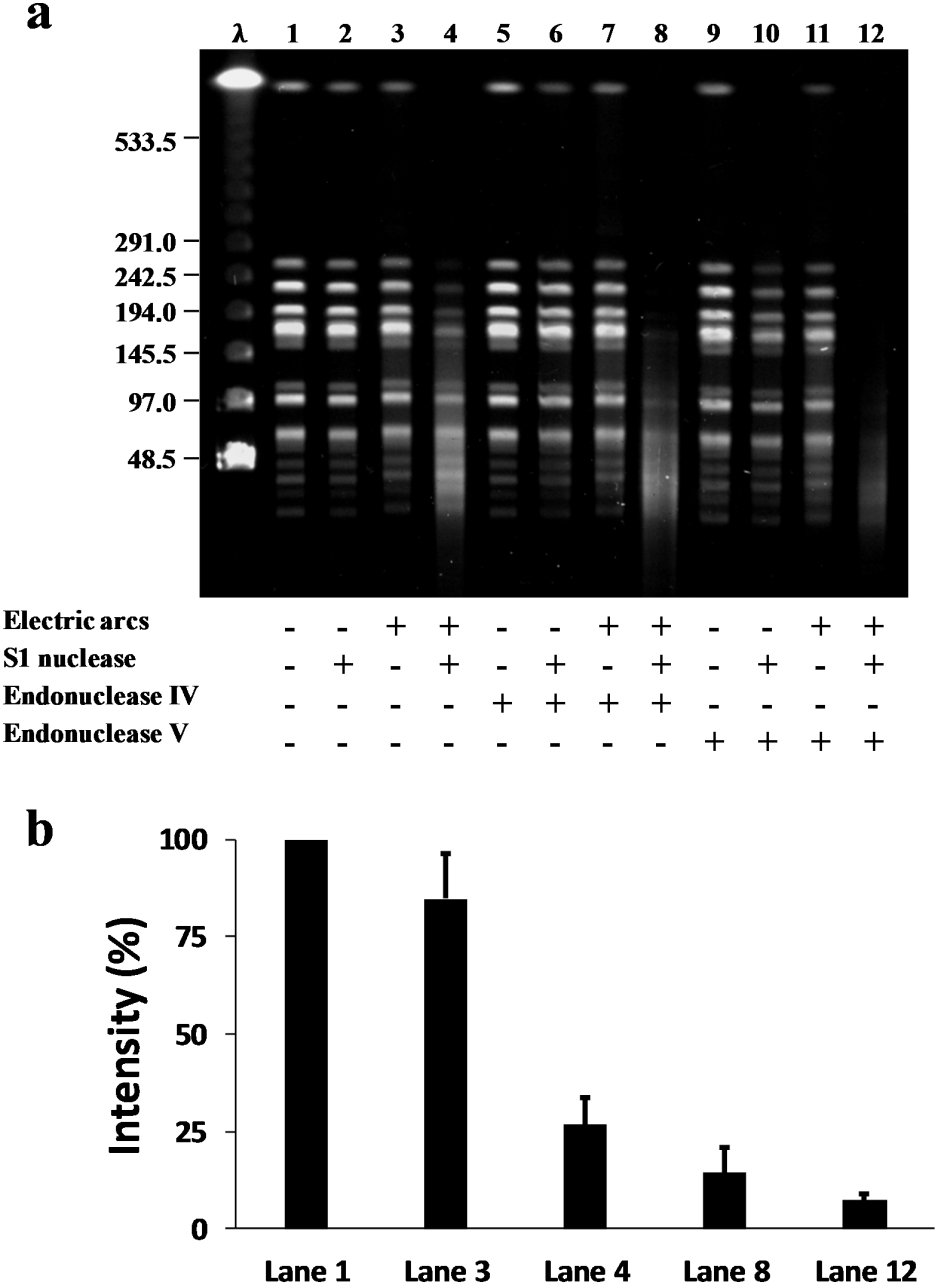
Electric arcs induce single-strand DNA breaks, base excisions and pyrimidines dimers. **(a)** Pulsed-Field Gel electrophoresis (PFGE) patterns of *NotI*-digested chromosomal DNA (double-strand) from *Bacillus pumilus* spores exposed (+) or not (-) to electric arcs. The DNA migration was observed with (+) and without (-) various endonuclease enzymes. The S1 nuclease cleaves the single-strand DNA; the endonuclease IV cleaves the apurinic/apyrimidinic sites; the endonuclease V cleaves the pyrimidine dimers. The size range of the standard Lambda Ladder is between 48.5 and 1000 kb. In absence of electric arcs (lanes 1, 2, 5, 6, 9, 11), DNA was intact. After electric arcs exposure; the migration of fragments was slowed down (lane 3); the S1 nuclease revealed single-strand DNA breaks (lane 4); the combination with the endonucleases S1 and IV exhibited base excisions (lane 8); the combination with the endonucleases S1 and V showed the presence of pyrimidine dimers (lane 12). **(b)** Example of quantification of the fluorescence intensity (in percentage compared to the control, lane 1) on the higher fragment (between 242.5 and 291 kb), calculated from 3 independent experiments. Lane 1: control; Lane 3: loss of intensity of 15% with electric arcs alone; Lane 4: loss of 75% with electric arcs and S1 nuclease; Lane 8: loss of 85% with electric arcs and S1 nuclease combined with endonuclease IV; Lane 12: loss of 93% with electric arcs and S1 nuclease combined with endonuclease V.

The integrity of chromosomal DNA was analyzed after electric arcs exposure by the use of PFGE (Fig 5). *NotI*-digested fragments (double-stranded) of chromosomal DNA were extracted from *B*. *pumilus* spores and analyzed after migration by PFGE. In order to identify DNA damage, various endonucleases were used. The endonuclease IV and T4 endonuclease V induce single-strand DNA break at the 5’ apurinic/apyrimidinic sites and pyrimidine dimers, respectively. To quantify single-strand DNA breaks, the S1 endonuclease was used. This enzyme specifically cleaves single-stranded regions to form double-strand breaks which can be detected by PFGE [34–36].

We first evaluated the DNA damage induced by electric arcs exposure (Fig 5). In control condition, *NotI*-digested fragments were clearly distinguished (lane 1). Similar patterns were observed after incubation with the endonucleases S1, IV and V (lanes 2, 5, 6, 9 and 10). These results confirmed the absence of DNA damage for the unexposed spores. After electric arcs treatment, the migration of fragments was slowed down (lane 3). The S1 endonuclease induced the degradation of the *NotI*-digested fragments and the formation of a smear (lane 4) with a lower intensity of 75% (Fig 5b) for the higher fragment. These observations revealed the presence of single-strand DNA breaks. The incubations with the endonucleases IV and V without S1 (lanes 7 and 11) were used as control and no significant degradation was observed. In presence of the S1 nuclease combined with the endonuclease IV, a large smear was observed (lane 8) with a loss of intensity of 85%. This result revealed the presence of bases excised during electric arcs exposure. After incubation with the S1 nuclease and the endonuclease V, the DNA fragments were much more degraded (lane 12) with a loss of intensity of 93%. Thus, the presence of pyrimidine dimers was exhibited after electric arcs exposure.

## Discussion

After electric arcs exposure, morphological imaging by SEM did not reveal any variation of spore size and the protein ridges remained intact on the coat surface. AFM in liquid validated these observations at the nanoscale and no variation of roughness was observed. TEM images confirmed the preservation of the spore structure and the visible integrity of the spores. These results demonstrated a non-physical destruction of spores at the nanometric scale by the electric arcs. In the literature, the shock waves are currently described as an efficient method to inactivate bacteria [37,38] by a mechanical disruption of the bacterial cell wall [14]. Furthermore, in a previous work we demonstrated that the disorganization of the spore structure is caused by Pulsed Electric Fields (PEF) [21]. Here, the absence of visible damage on the spore structure, suggested the non-implication of PEF during electric arcs exposure. In conclusion, the inactivation of spores during electric arcs treatment was not due to electric fields nor to shock waves. However, the possible presence of cell wall damage in vegetative bacteria suggested that more vulnerable microorganisms than spores could be impacted by electric fields and shock waves.

In the absence of mechanical disruption of spores, the incidence of electric arcs on the chromosomal DNA was considered because we demonstrated an incidence of UVs and ROS in spore inactivation during electric arcs exposure. PFGE experiments revealed a high occurrence of single-strand DNA breaks, base excisions and pyrimidine dimers. Firstly, the abundance of the observed base excisions by PFGE suggested the production of ROS during electric arcs exposure. The ROS are classically considered as very efficient to kill microorganisms [13,39] by the production of DNA damage, including base modifications and DNA strand breaks [20,40]. The presence of base excisions after electric arcs exposure confirmed the role of ROS in base excisions. Secondly, UVs are known to induce mutagenic lesions in DNA, particularly DNA strand breaks and pyrimidine dimers [18]. The abundance of pyrimidine dimers after electric arcs exposure confirmed the implication of UV radiation in bacterial inactivation. In addition, the slow migration of DNA fragments may be explained by DNA bend induced by pyrimidine dimers [41].

## Conclusion

**Fig 6:**
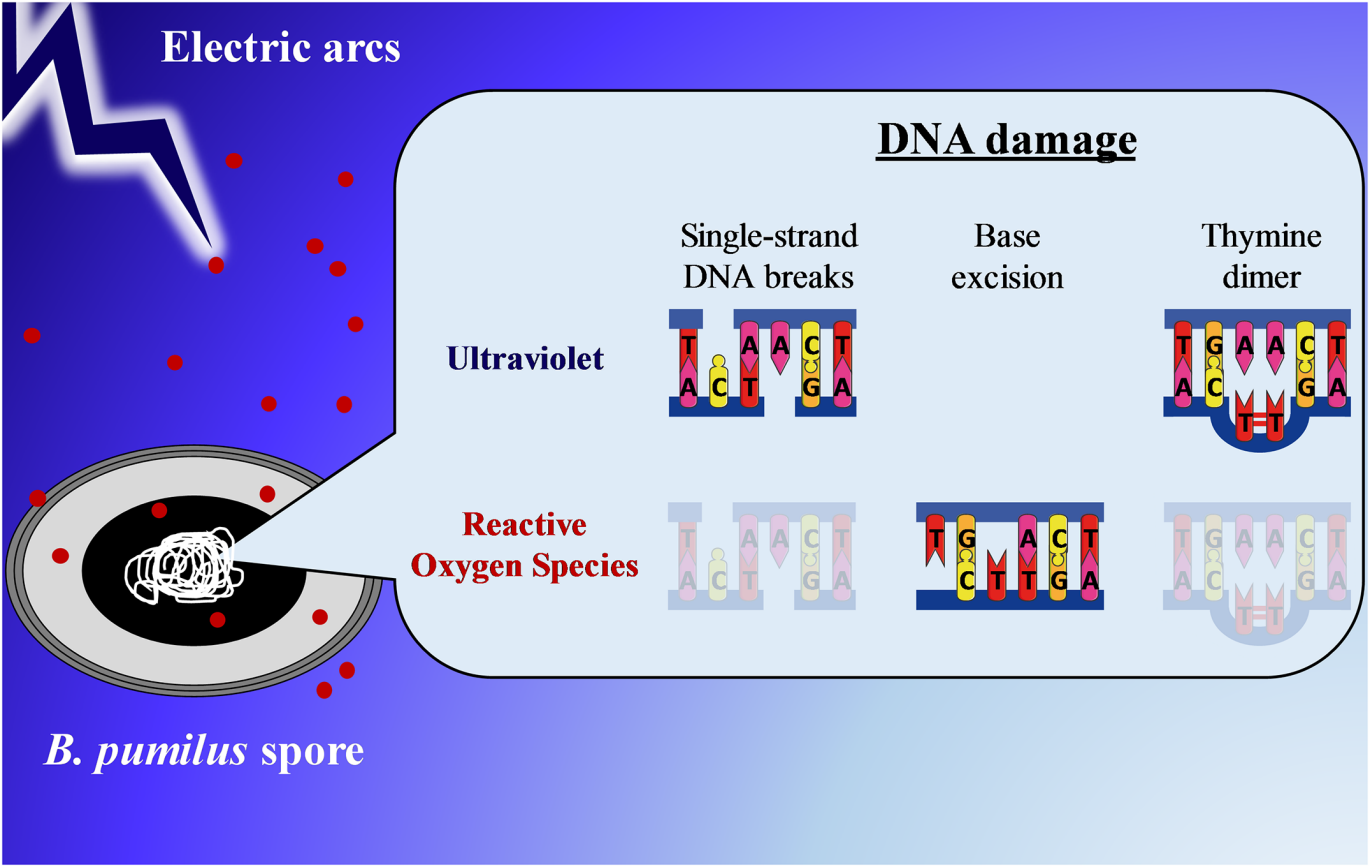
Schematic illustration of the phenomenon involved in spore inactivation during electric arcs exposure. The core contained DNA supercoiled, SASPs, water, dipicolinic acids and Ca^2+^. The morphology and the spore structure were not affected during electric arcs exposure. Many DNA damage were observed after spore inactivation. UV radiation induced single-strand DNA breaks and pyrimidine dimers formation (thymine or cytosine dimers). Reactive Oxygen Species (ROS) were mainly implicated in base excision and moderately involved in single-strand DNA breaks and thymine dimer. Red dots represent ROS.

To our knowledge, we are the first to explore the mechanisms of spore inactivation upon electric arcs exposure in water. This new and innovative technology to kill bacteria in suspension could be promising for the sterilization of cooling towers and canalizations. We used a multidisciplinary approach to reveal the cause(s) of spore inactivation from the single-cell to the molecular level. We explored the spore structure by cutting-edge microscopies, as well as the integrity of the genomic DNA. We found that the inactivation is not due to physical damage caused by shock waves or electric field exposure but the consequence of molecular alterations. We demonstrated here chromosomal damage induced by electric arcs with a significant implication of UV and ROS phenomena (Fig 6). This work allowed to get access for the first time to the consequences of electric arcs exposure on spore structure and chromosomal DNA. For a complete understanding of the overall mechanisms involved, it would be interesting to explore the incidence of electric arcs on other biomolecules such as proteins.

## Supporting information

S1 Fig Principle of electric arcs generation. An increasing current is applied in water with an inter-electrode distance of 2 mm. The formation of electric arcs is initiated in three steps. **(a)** Thermal increase. At low current, the electric field induces a joule heating between the electrodes. **(b)** Bubble formation. When the voltage increases, a part of water is evaporated and some little bubbles appear. **(c)** Electric arc discharge. At 120 kV, the bubbles grow until became large enough to initiate breakdown and an electric arc between the electrodes.
(DOCX)

S2 Fig Evolution of the number of photons created in function of the number of pulses. This experiment was made in triplicate.
(DOCX)

S3 Fig Detection of single-strand breaks, base excisions and pyrimidine dimers damage. **(a)** Detection of single-strand breaks. At low concentration, the S1 nuclease specifically recognizes single-strand breaks and converts them into double-strand breaks. **(b)** Detection of base excisions. The endonuclease IV specifically recognizes the apurinic/apyrimidinic sites (AP sites) and cleaves the phosphodiester bond in 5’ to generate a single-strand DNA break. The S1 nuclease cleaves the single-stranded regions to form double-strand breaks. **(c)** Detection of pyrimidine dimers. The T4 endonuclease V has both activities. First, the enzyme recognizes pyrimidine dimers and cleaves the glycosyl bond at the 5’ end of the damage. Second, the endonucleolytic activity cleaves the phosphodiester bond at the AP site to generate a single-strand break. The S1 nuclease cleaves the single-stranded sites to form double-strand breaks.
(DOCX)

S4 Fig Effect of ROS and UV radiation on the *Bacillus pumilus* spores after 500 electric arcs exposure. After 500 arcs in distilled water, an inactivation rate of 2.2 log_10_ ± 0.2 (99%) was observed (dark bar). In presence of 1 mM pyruvate to neutralize the ROS, an inactivation rate of 1.78 log_10_ ± 0.2 (98%) was obtained (grey bar). In presence of UVs alone (without ROS, shock waves and electric field), the inactivation rate was 0.72 log_10_ ± 0.08 (81%) (bar hachured). This experiment was made in triplicate. Statistical analysis were performed using the t-test (***P < 0.001).
(DOCX)

S5 Fig Electric arcs induce cytoplasm leakage on vegetative bacteria *Bacillus pumilus*. The inactivation rate was 2.55 log_10_ after 500 electric arcs. **(a)** TEM images showing example of untreated vegetative bacteria. Cell wall (CW), plasma membrane (PM) and cytoplasm (Cy) were undamaged. **(b)** Vegetative bacteria were visualized after electric arcs exposure. The presence of inclusion bodies (IB) was a consequence of the cytoplasm content leakage probably due to a cell wall disturbance. Scale bars: 100 nm. At least, 10 cells were imaged for each condition and one representative image was shown.
(DOCX)

## Abbreviations

64PP: (6–4)-PhotoProduct
AFM: Atomic Force Microscopy
CPD: Cyclobutane Pyrimidine Dimer
DPA: DiPicolinic Acid
PCA: Plate Count Agar
PEF: Pulsed Electric Field
PFGE: Pulsed-Field Gel Electrophoresis
ROS: Reactive Oxygen Species
SASPs: Small, Acid-Soluble spore Proteins
SEM: Scanning Electron Microscopy
SP: Spore Photoproduct
TEM: Transmission Electron Microscopy
