# Supplementary material for: Electrical discharges in water induce spores’ DNA damage
Source: PLoS One. 2018 Aug 13;13(8):e0201448. doi: 10.1371/journal.pone.0201448 (PMC6089432; doi:10.1371/journal.pone.0201448)
Supplement: S1 Fig — An increasing current is applied in water with an inter-electrode distance of 2 mm. The formation of electric arcs is initiated in three steps. (a) Thermal increase. At low current, the electric field induces a joule heating between the electrodes. (b) Bubble formation. When the voltage increases, a part of water is evaporated and some little bubbles appear. (c) Electric arc discharge. At 120 kV, the bubbles grow until became large enough to initiate breakdown and an electric arc between the electrodes. (DOCX) [file pone.0201448.s001.docx]

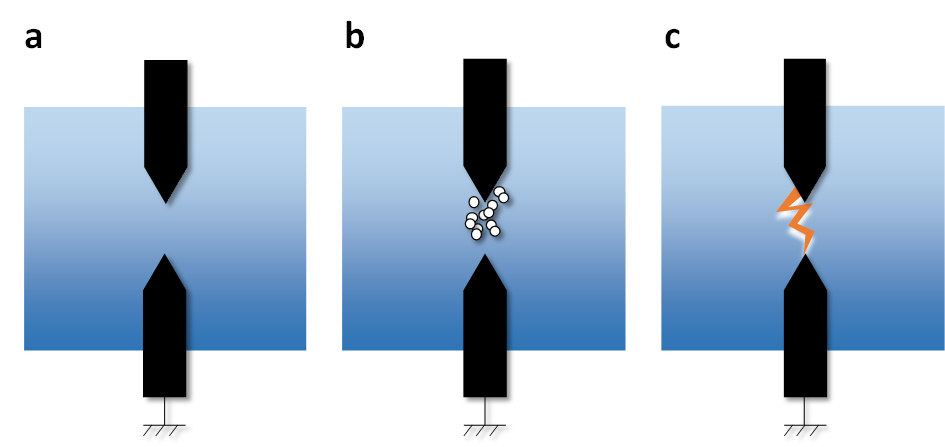


**S1 Fig: Principle of electric arcs generation.** An increasing current is applied in water with an inter-electrode distance of 2 mm. The formation of electric arcs is initiated in three steps. **(a)** Thermal increase. At low current, the electric field induces a joule heating between the electrodes. **(b)** Bubble formation. When the voltage increases, a part of water is evaporated and some little bubbles appear. **(c)** Electric arc discharge. At 120 kV, the bubbles grow until became large enough to initiate breakdown and an electric arc between the electrodes.
