# Supplementary material for: Electrical discharges in water induce spores’ DNA damage
Source: PLoS One. 2018 Aug 13;13(8):e0201448. doi: 10.1371/journal.pone.0201448 (PMC6089432; doi:10.1371/journal.pone.0201448)
Supplement: S2 Fig — This experiment was made in triplicate. (DOCX) [file pone.0201448.s002.docx]

**S2 Fig: Evolution of the number of photons created in function of the number of pulses.** This experiment was made in triplicate.
