# Supplementary material for: Electrical discharges in water induce spores’ DNA damage
Source: PLoS One. 2018 Aug 13;13(8):e0201448. doi: 10.1371/journal.pone.0201448 (PMC6089432; doi:10.1371/journal.pone.0201448)
Supplement: S3 Fig — (a) Detection of single-strand breaks. At low concentration, the S1 nuclease specifically recognizes single-strand breaks and converts them into double-strand breaks. (b) Detection of base excisions. The endonuclease IV specifically recognizes the apurinic/apyrimidinic sites (AP sites) and cleaves the phosphodiester bond in 5’ to generate a single-strand DNA break. The S1 nuclease cleaves the single-stranded regions to form double-strand breaks. (c) Detection of pyrimidine dimers. The T4 endonuclease V has both activities. First, the enzyme recognizes pyrimidine dimers and cleaves the glycosyl bond at the 5’ end of the damage. Second, the endonucleolytic activity cleaves the phosphodiester bond at the AP site to generate a single-strand break. The S1 nuclease cleaves the single-stranded sites to form double-strand breaks. (DOCX) [file pone.0201448.s003.docx]

**S3 Fig: Detection of single-strand breaks, base excisions and pyrimidine dimers damage. (a)** Detection of single-strand breaks. At low concentration, the S1 nuclease specifically recognizes single-strand breaks and converts them into double-strand breaks.  **(b)** Detection of base excisions. The endonuclease IV specifically recognizes the apurinic/apyrimidinic sites (AP sites) and cleaves the phosphodiester bond in 5’ to generate a single-strand DNA break. The S1 nuclease cleaves the single-stranded regions to form double-strand breaks. **(c)** Detection of pyrimidine dimers. The T4 endonuclease V has both activities. First, the enzyme recognizes pyrimidine dimers and cleaves the glycosyl bond at the 5’ end of the damage. Second, the endonucleolytic activity cleaves the phosphodiester bond at the AP site to generate a single-strand break. The S1 nuclease cleaves the single-stranded sites to form double-strand breaks.
