# Supplementary material for: Electrical discharges in water induce spores’ DNA damage
Source: PLoS One. 2018 Aug 13;13(8):e0201448. doi: 10.1371/journal.pone.0201448 (PMC6089432; doi:10.1371/journal.pone.0201448)
Supplement: S4 Fig — After 500 arcs in distilled water, an inactivation rate of 2.2 log10 ± 0.2 (99%) was observed (dark bar). In presence of 1 mM pyruvate to neutralize the ROS, an inactivation rate of 1.78 log10 ± 0.2 (98%) was obtained (grey bar). In presence of UVs alone (without ROS, shock waves and electric field), the inactivation rate was 0.72 log10 ± 0.08 (81%) (bar hachured). This experiment was made in triplicate. Statistical analysis were performed using the t-test (***P < 0.001). (DOCX) [file pone.0201448.s004.docx]

**S4 Fig: Effect of ROS and UV radiation on the *Bacillus pumilus* spores after 500 electric arcs exposure.** After 500 arcs in distilled water, an inactivation rate of 2.2 log_10_ ± 0.2 (99%) was observed (dark bar). In presence of 1 mM pyruvate to neutralize the ROS, an inactivation rate of 1.78 log_10_ ± 0.2 (98%) was obtained (grey bar). In presence of UVs alone (without ROS, shock waves and electric field), the inactivation rate was 0.72 log_10_ ± 0.08 (81%) (bar hachured). This experiment was made in triplicate. Statistical analysis were performed using the t-test (***P < 0.001).
