# Supplementary material for: Electrical discharges in water induce spores’ DNA damage
Source: PLoS One. 2018 Aug 13;13(8):e0201448. doi: 10.1371/journal.pone.0201448 (PMC6089432; doi:10.1371/journal.pone.0201448)
Supplement: S5 Fig — The inactivation rate was 2.55 log10 after 500 electric arcs. (a) TEM images showing example of untreated vegetative bacteria. Cell wall (CW), plasma membrane (PM) and cytoplasm (Cy) were undamaged. (b) Vegetative bacteria were visualized after electric arcs exposure. The presence of inclusion bodies (IB) was a consequence of the cytoplasm content leakage probably due to a cell wall disturbance. Scale bars: 100 nm. At least, 10 cells were imaged for each condition and one representative image was shown. (DOCX) [file pone.0201448.s005.docx]

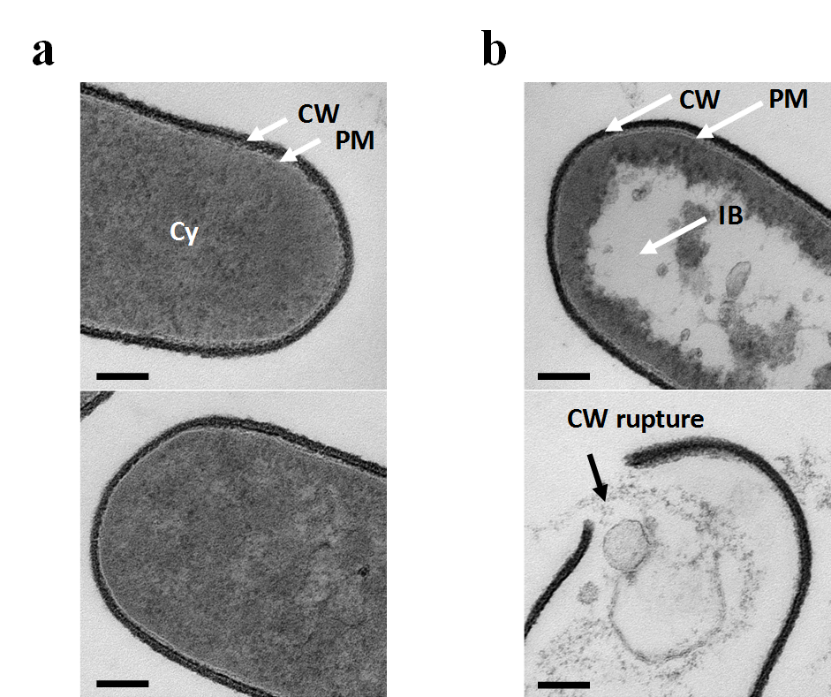


**S5 Fig: Electric arcs induce cytoplasm leakage on vegetative bacteria *Bacillus pumilus*.** The inactivation rate was 2.55 log_10_ after 500 electric arcs. **(a)** TEM images showing example of untreated vegetative bacteria. Cell wall (CW), plasma membrane (PM) and cytoplasm (Cy) were undamaged. **(b)** Vegetative bacteria were visualized after electric arcs exposure. The presence of inclusion bodies (IB) was a consequence of the cytoplasm content leakage probably due to a cell wall disturbance. Scale bars: 100 nm. At least, 10 cells were imaged for each condition and one representative image was shown.
